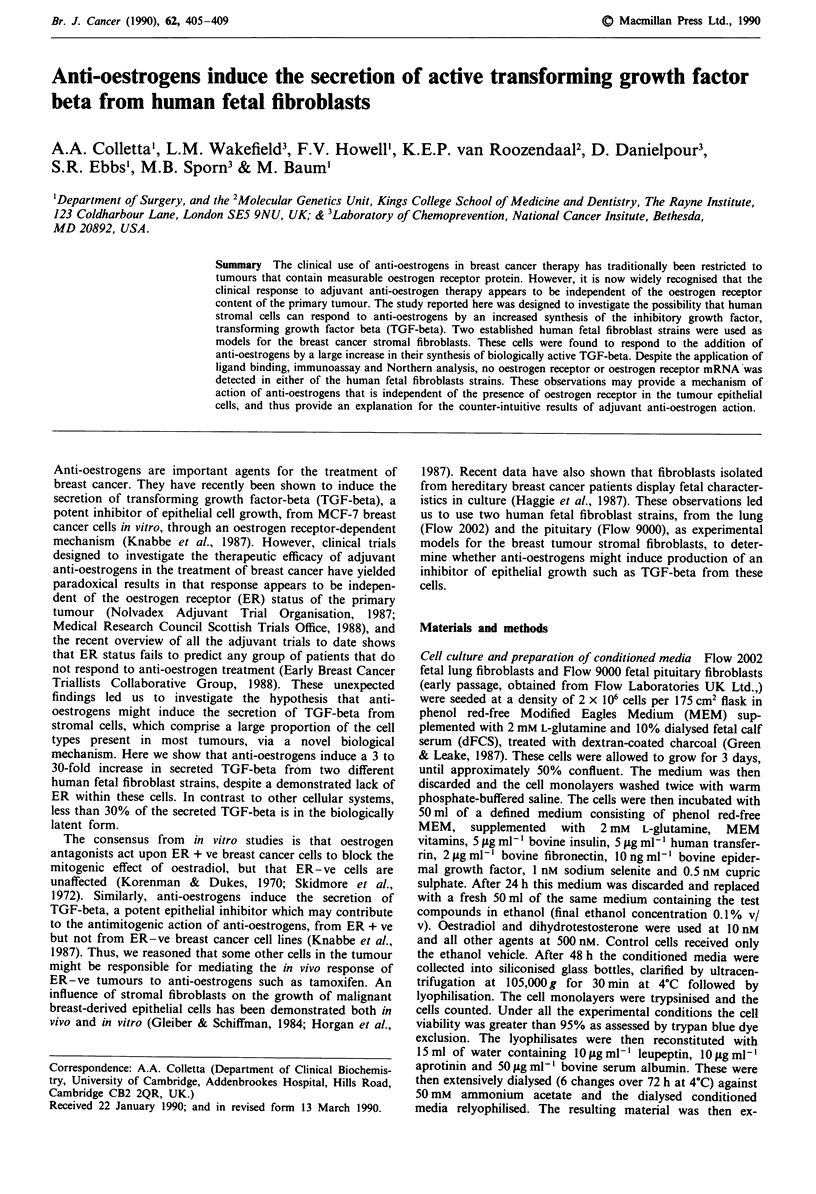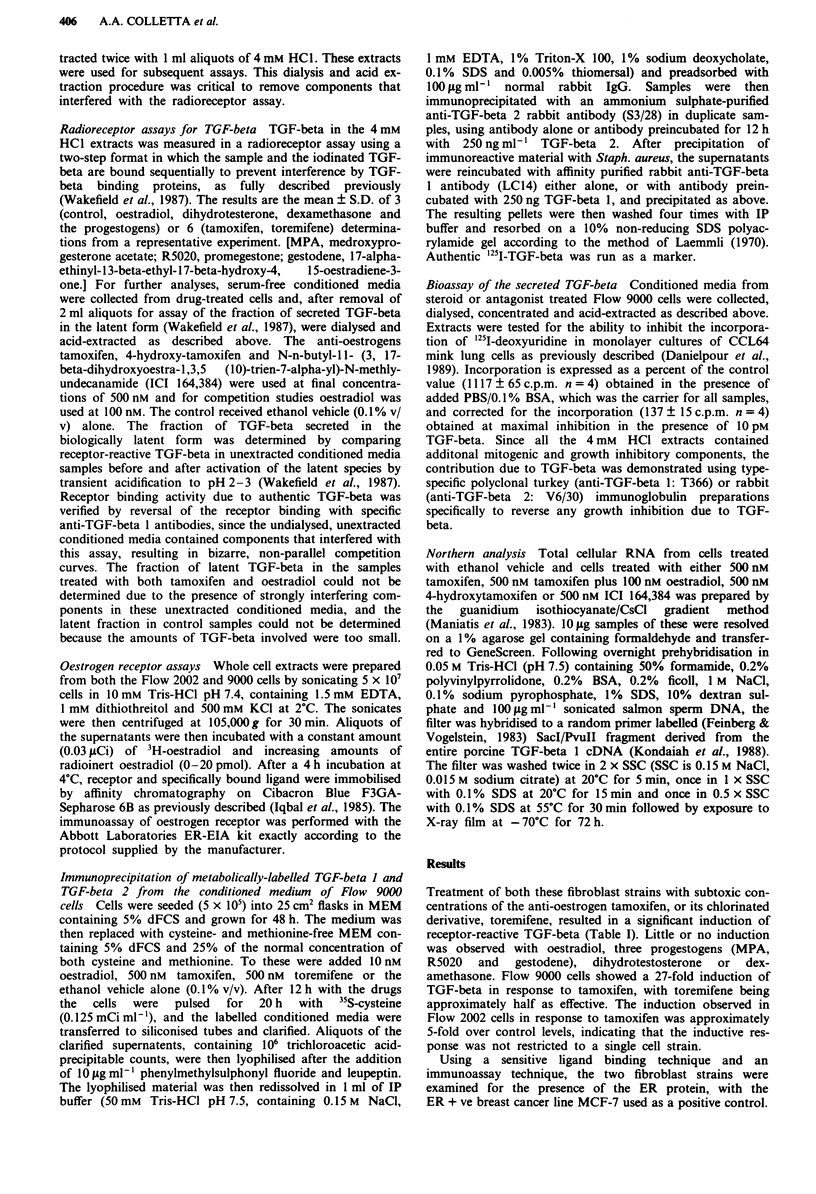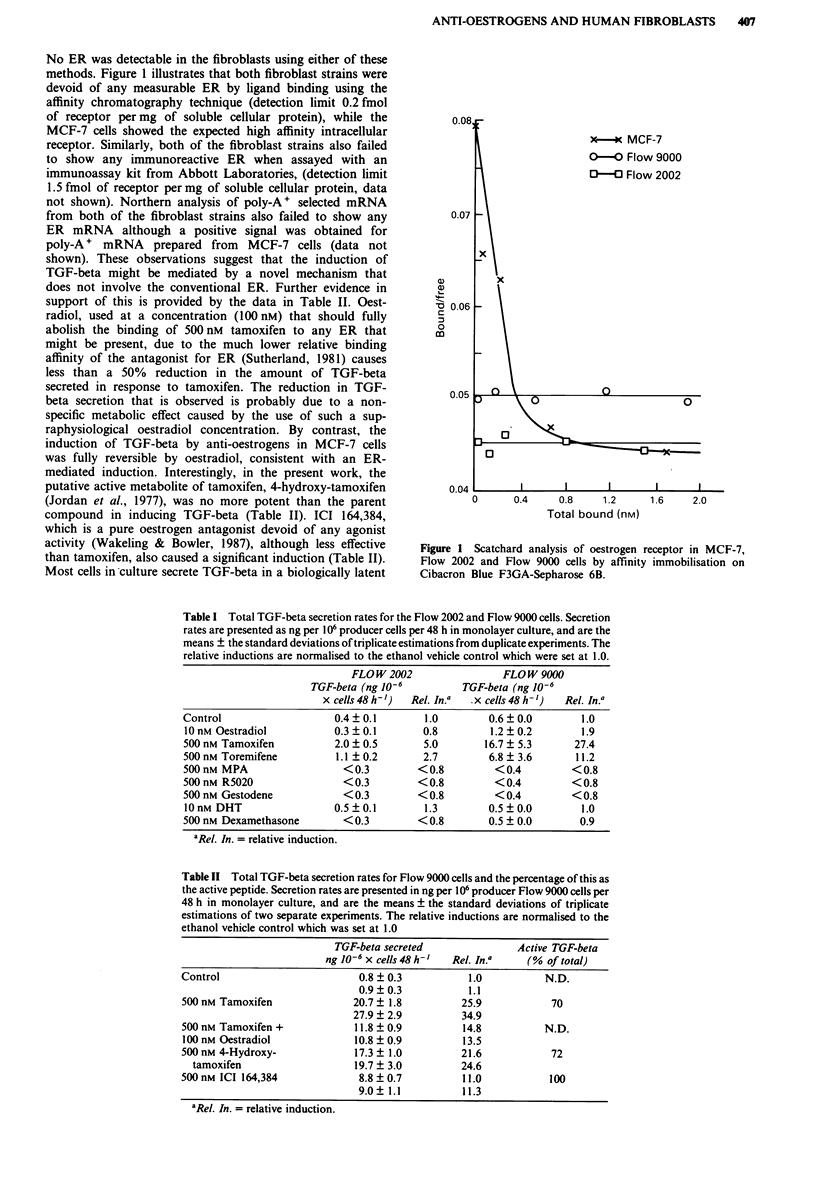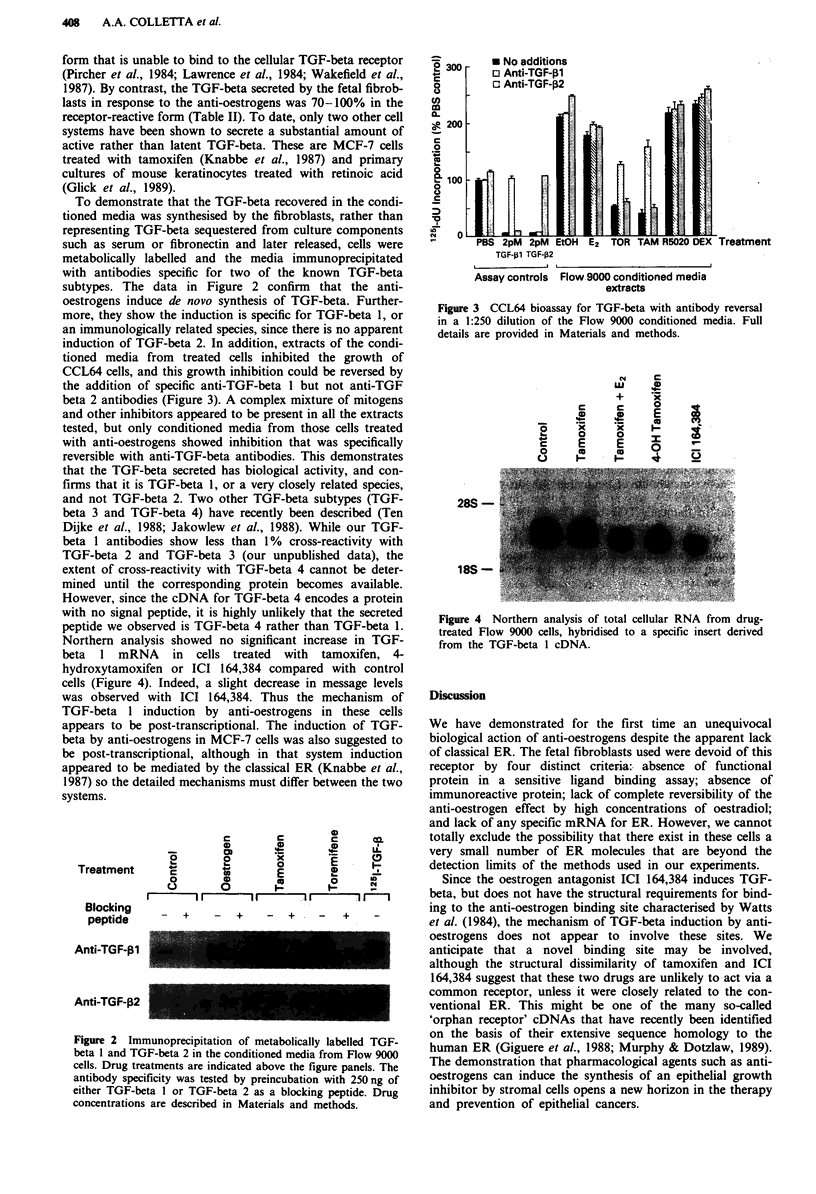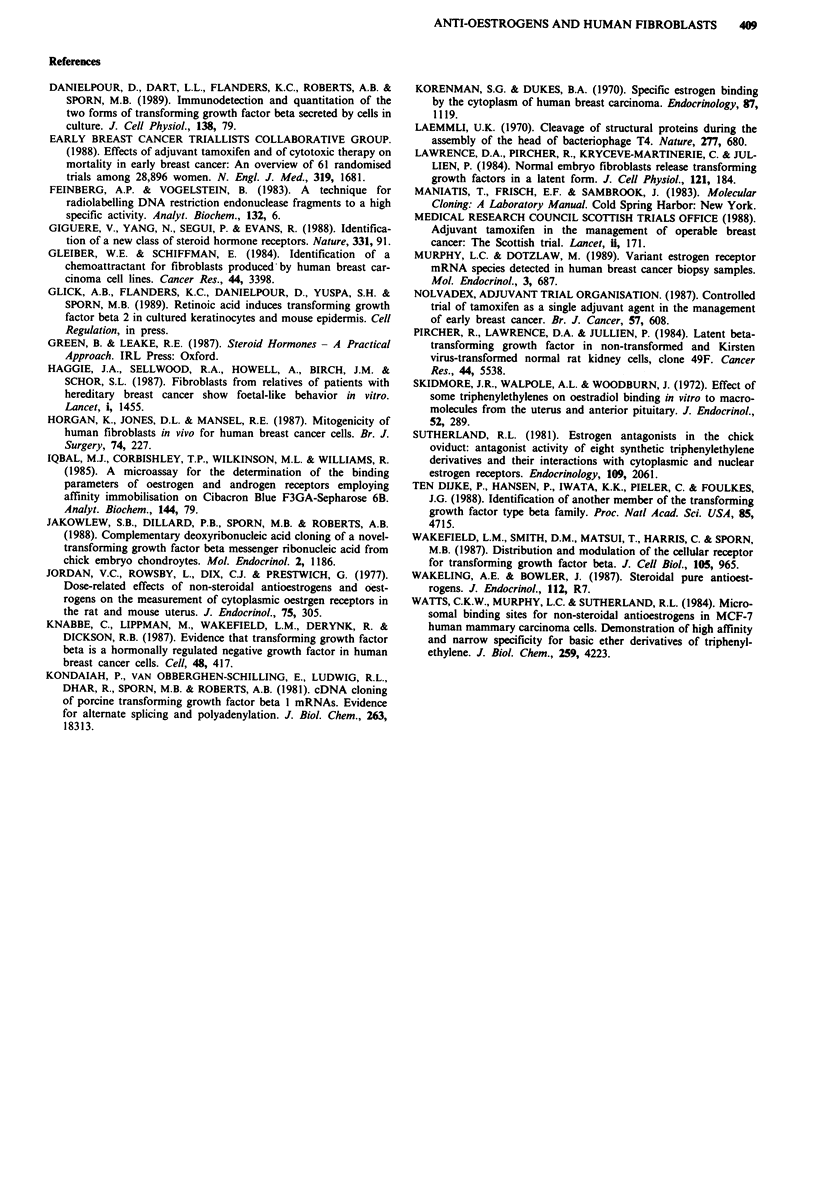# Anti-oestrogens induce the secretion of active transforming growth factor beta from human fetal fibroblasts.

**DOI:** 10.1038/bjc.1990.307

**Published:** 1990-09

**Authors:** A. A. Colletta, L. M. Wakefield, F. V. Howell, K. E. van Roozendaal, D. Danielpour, S. R. Ebbs, M. B. Sporn, M. Baum

**Affiliations:** Department of Surgery, Kings College School of Medicine and Dentistry, Rayne Institute, London, UK.

## Abstract

**Images:**


Br. J. Cancer (1990), 62, 405-409 © Macmillan Press Ltd., 1990

### Anti-oestrogens induce the secretion of active transforming growth factor beta from human fetal fibroblasts

A.A. Colletta[1], L.M. Wakefield[3], F.V. Howell[1], K.E.P. van Roozendaal[2], D. Danielpour[3], S.R. Ebbs[1], M.B. Sporn[3] & M. Baum[1]

[1]*Department of Surgery, and the* [2]*Molecular Genetics Unit, Kings College School of Medicine and Dentistry, The Rayne Institute, 123 Coldharbour Lane, London SE5 9NU, UK;* & [3]*Laboratory of Chemoprevention, National Cancer Insitute, Bethesda, MD 20892, USA.*

**Summary** The clinical use of anti-oestrogens in breast cancer therapy has traditionally been restricted to tumours that contain measurable oestrogen receptor protein. However, it is now widely recognised that the clinical response to adjuvant anti-oestrogen therapy appears to be independent of the oestrogen receptor content of the primary tumour. The study reported here was designed to investigate the possibility that human stromal cells can respond to anti-oestrogens by an increased synthesis of the inhibitory growth factor, transforming growth factor beta (TGF-beta). Two established human fetal fibroblast strains were used as models for the breast cancer stromal fibroblasts. These cells were found to respond to the addition of anti-oestrogens by a large increase in their synthesis of biologically active TGF-beta. Despite the application of ligand binding, immunoassay and Northern analysis, no oestrogen receptor or oestrogen receptor mRNA was detected in either of the human fetal fibroblasts strains. These observations may provide a mechanism of action of anti-oestrogens that is independent of the presence of oestrogen receptor in the tumour epithelial cells, and thus provide an explanation for the counter-intuitive results of adjuvant anti-oestrogen action.

Anti-oestrogens are important agents for the treatment of breast cancer. They have recently been shown to induce the secretion of transforming growth factor-beta (TGF-beta), a potent inhibitor of epithelial cell growth, from MCF-7 breast cancer cells *in vitro*, through an oestrogen receptor-dependent mechanism (Knabbe *et al*., 1987). However, clinical trials designed to investigate the therapeutic efficacy of adjuvant anti-oestrogens in the treatment of breast cancer have yielded paradoxical results in that response appears to be independent of the oestrogen receptor (ER) status of the primary tumour (Nolvadex Adjuvant Trial Organisation, 1987; Medical Research Council Scottish Trials Office, 1988), and the recent overview of all the adjuvant trials to date shows that ER status fails to predict any group of patients that do not respond to anti-oestrogen treatment (Early Breast Cancer Triallists Collaborative Group, 1988). These unexpected findings led us to investigate the hypothesis that anti-oestrogens might induce the secretion of TGF-beta from stromal cells, which comprise a large proportion of the cell types present in most tumours, via a novel biological mechanism. Here we show that anti-oestrogens induce a 3 to 30-fold increase in secreted TGF-beta from two different human fetal fibroblast strains, despite a demonstrated lack of ER within these cells. In contrast to other cellular systems, less than 30% of the secreted TGF-beta is in the biologically latent form.

The consensus from *in vitro* studies is that oestrogen antagonists act upon ER + ve breast cancer cells to block the mitogenic effect of oestradiol, but that ER-ve cells are unaffected (Korenman & Dukes, 1970; Skidmore *et al*., 1972). Similarly, anti-oestrogens induce the secretion of TGF-beta, a potent epithelial inhibitor which may contribute to the antimitogenic action of anti-oestrogens, from ER + ve but not from ER-ve breast cancer cell lines (Knabbe *et al*., 1987). Thus, we reasoned that some other cells in the tumour might be responsible for mediating the *in vivo* response of ER-ve tumours to anti-oestrogens such as tamoxifen. An influence of stromal fibroblasts on the growth of malignant breast-derived epithelial cells has been demonstrated both *in vivo* and *in vitro* (Gleiber & Schiffman, 1984; Horgan *et al*., 1987). Recent data have also shown that fibroblasts isolated from hereditary breast cancer patients display fetal characteristics in culture (Haggie *et al*., 1987). These observations led us to use two human fetal fibroblast strains, from the lung (Flow 2002) and the pituitary (Flow 9000), as experimental models for the breast tumour stromal fibroblasts, to determine whether anti-oestrogens might induce production of an inhibitor of epithelial growth such as TGF-beta from these cells.

Correspondence: A.A. Colletta (Department of Clinical Biochemistry, University of Cambridge, Addenbrookes Hospital, Hills Road, Cambridge CB2 2QR, UK.)
Received 22 January 1990; and in revised form 13 March 1990.

### Materials and methods

*Cell culture and preparation of conditioned media* Flow 2002 fetal lung fibroblasts and Flow 9000 fetal pituitary fibroblasts (early passage, obtained from Flow Laboratories UK Ltd.,) were seeded at a density of $2 \times 10^6$ cells per 175 $cm^2$ flask in phenol red-free Modified Eagles Medium (MEM) supplemented with 2 mM L-glutamine and 10% dialysed fetal calf serum (dFCS), treated with dextran-coated charcoal (Green & Leake, 1987). These cells were allowed to grow for 3 days, until approximately 50% confluent. The medium was then discarded and the cell monolayers washed twice with warm phosphate-buffered saline. The cells were then incubated with 50 ml of a defined medium consisting of phenol red-free MEM, supplemented with 2 mM L-glutamine, MEM vitamins, 5 $\mu g\ ml^{-1}$ bovine insulin, 5 $\mu g\ ml^{-1}$ human transferrin, 2 $\mu g\ ml^{-1}$ bovine fibronectin, 10 $ng\ ml^{-1}$ bovine epidermal growth factor, 1 nM sodium selenite and 0.5 nM cupric sulphate. After 24 h this medium was discarded and replaced with a fresh 50 ml of the same medium containing the test compounds in ethanol (final ethanol concentration 0.1% v/v). Oestradiol and dihydrotestosterone were used at 10 nM and all other agents at 500 nM. Control cells received only the ethanol vehicle. After 48 h the conditioned media were collected into siliconised glass bottles, clarified by ultracentrifugation at 105,000 *g* for 30 min at 4°C followed by lyophilisation. The cell monolayers were trypsinised and the cells counted. Under all the experimental conditions the cell viability was greater than 95% as assessed by trypan blue dye exclusion. The lyophilisates were then reconstituted with 15 ml of water containing 10 $\mu g\ ml^{-1}$ leupeptin, 10 $\mu g\ ml^{-1}$ aprotinin and 50 $\mu g\ ml^{-1}$ bovine serum albumin. These were then extensively dialysed (6 changes over 72 h at 4°C) against 50 mM ammonium acetate and the dialysed conditioned media relyophilised. The resulting material was then extracted twice with 1 ml aliquots of 4 mM HCl. These extracts were used for subsequent assays. This dialysis and acid extraction procedure was critical to remove components that interfered with the radioreceptor assay.

*Radioreceptor assays for TGF-beta* TGF-beta in the 4 mM HCl extracts was measured in a radioreceptor assay using a two-step format in which the sample and the iodinated TGF-beta are bound sequentially to prevent interference by TGF-beta binding proteins, as fully described previously (Wakefield *et al.*, 1987). The results are the mean ± S.D. of 3 (control, oestradiol, dihydrotestosterone, dexamethasone and the progestogens) or 6 (tamoxifen, toremifene) determinations from a representative experiment. [MPA, medroxyprogesterone acetate; R5020, promegestone; gestodene, 17-alpha-ethinyl-13-beta-ethyl-17-beta-hydroxy-4, 15-oestradiene-3-one.] For further analyses, serum-free conditioned media were collected from drug-treated cells and, after removal of 2 ml aliquots for assay of the fraction of secreted TGF-beta in the latent form (Wakefield *et al.*, 1987), were dialysed and acid-extracted as described above. The anti-oestrogens tamoxifen, 4-hydroxy-tamoxifen and N-n-butyl-11- (3, 17-beta-dihydroxyoestra-1,3,5 (10)-trien-7-alpha-yl)-N-methly-undecanamide (ICI 164,384) were used at final concentrations of 500 nM and for competition studies oestradiol was used at 100 nM. The control received ethanol vehicle (0.1% v/v) alone. The fraction of TGF-beta secreted in the biologically latent form was determined by comparing receptor-reactive TGF-beta in unextracted conditioned media samples before and after activation of the latent species by transient acidification to pH 2–3 (Wakefield *et al.*, 1987). Receptor binding activity due to authentic TGF-beta was verified by reversal of the receptor binding with specific anti-TGF-beta 1 antibodies, since the undialysed, unextracted conditioned media contained components that interfered with this assay, resulting in bizarre, non-parallel competition curves. The fraction of latent TGF-beta in the samples treated with both tamoxifen and oestradiol could not be determined due to the presence of strongly interfering components in these unextracted conditioned media, and the latent fraction in control samples could not be determined because the amounts of TGF-beta involved were too small.

*Oestrogen receptor assays* Whole cell extracts were prepared from both the Flow 2002 and 9000 cells by sonicating $5 \times 10^7$ cells in 10 mM Tris-HCl pH 7.4, containing 1.5 mM EDTA, 1 mM dithiothreitol and 500 mM KCl at 2°C. The sonicates were then centrifuged at 105,000 *g* for 30 min. Aliquots of the supernatants were then incubated with a constant amount (0.03 μCi) of $^3$H-oestradiol and increasing amounts of radioinert oestradiol (0–20 pmol). After a 4 h incubation at 4°C, receptor and specifically bound ligand were immobilised by affinity chromatography on Cibacron Blue F3GA-Sepharose 6B as previously described (Iqbal *et al.*, 1985). The immunoassay of oestrogen receptor was performed with the Abbott Laboratories ER-EIA kit exactly according to the protocol supplied by the manufacturer.

*Immunoprecipitation of metabolically-labelled TGF-beta 1 and TGF-beta 2 from the conditioned medium of Flow 9000 cells* Cells were seeded ($5 \times 10^5$) into 25 cm$^2$ flasks in MEM containing 5% dFCS and grown for 48 h. The medium was then replaced with cysteine- and methionine-free MEM containing 5% dFCS and 25% of the normal concentration of both cysteine and methionine. To these were added 10 nM oestradiol, 500 nM tamoxifen, 500 nM toremifene or the ethanol vehicle alone (0.1% v/v). After 12 h with the drugs the cells were pulsed for 20 h with $^{35}$S-cysteine (0.125 mCi ml$^{-1}$), and the labelled conditioned media were transferred to siliconised tubes and clarified. Aliquots of the clarified supernatents, containing $10^6$ trichloroacetic acid-precipitable counts, were then lyophilised after the addition of 10 μg ml$^{-1}$ phenylmethylsulphonyl fluoride and leupeptin. The lyophilised material was then redissolved in 1 ml of IP buffer (50 mM Tris-HCl pH 7.5, containing 0.15 M NaCl, 1 mM EDTA, 1% Triton-X 100, 1% sodium deoxycholate, 0.1% SDS and 0.005% thiomersal) and preadsorbed with 100 μg ml$^{-1}$ normal rabbit IgG. Samples were then immunoprecipitated with an ammonium sulphate-purified anti-TGF-beta 2 rabbit antibody (S3/28) in duplicate samples, using antibody alone or antibody preincubated for 12 h with 250 ng ml$^{-1}$ TGF-beta 2. After precipitation of immunoreactive material with *Staph. aureus*, the supernatants were reincubated with affinity purified rabbit anti-TGF-beta 1 antibody (LC14) either alone, or with antibody preincubated with 250 ng TGF-beta 1, and precipitated as above. The resulting pellets were then washed four times with IP buffer and resorbed on a 10% non-reducing SDS polyacrylamide gel according to the method of Laemmli (1970). Authentic $^{125}$I-TGF-beta was run as a marker.

*Bioassay of the secreted TGF-beta* Conditioned media from steroid or antagonist treated Flow 9000 cells were collected, dialysed, concentrated and acid-extracted as described above. Extracts were tested for the ability to inhibit the incorporation of $^{125}$I-deoxyuridine in monolayer cultures of CCL64 mink lung cells as previously described (Danielpour *et al.*, 1989). Incorporation is expressed as a percent of the control value (1117 ± 65 c.p.m. $n = 4$) obtained in the presence of added PBS/0.1% BSA, which was the carrier for all samples, and corrected for the incorporation (137 ± 15 c.p.m. $n = 4$) obtained at maximal inhibition in the presence of 10 pM TGF-beta. Since all the 4 mM HCl extracts contained additonal mitogenic and growth inhibitory components, the contribution due to TGF-beta was demonstrated using type-specific polyclonal turkey (anti-TGF-beta 1: T366) or rabbit (anti-TGF-beta 2: V6/30) immunoglobulin preparations specifically to reverse any growth inhibition due to TGF-beta.

*Northern analysis* Total cellular RNA from cells treated with ethanol vehicle and cells treated with either 500 nM tamoxifen, 500 nM tamoxifen plus 100 nM oestradiol, 500 nM 4-hydroxytamoxifen or 500 nM ICI 164,384 was prepared by the guanidium isothiocyanate/CsCl gradient method (Maniatis *et al.*, 1983). 10 μg samples of these were resolved on a 1% agarose gel containing formaldehyde and transferred to GeneScreen. Following overnight prehybridisation in 0.05 M Tris-HCl (pH 7.5) containing 50% formamide, 0.2% polyvinylpyrrolidone, 0.2% BSA, 0.2% ficoll, 1 M NaCl, 0.1% sodium pyrophosphate, 1% SDS, 10% dextran sulphate and 100 μg ml$^{-1}$ sonicated salmon sperm DNA, the filter was hybridised to a random primer labelled (Feinberg & Vogelstein, 1983) SacI/PvuII fragment derived from the entire porcine TGF-beta 1 cDNA (Kondaiah *et al.*, 1988). The filter was washed twice in 2 × SSC (SSC is 0.15 M NaCl, 0.015 M sodium citrate) at 20°C for 5 min, once in 1 × SSC with 0.1% SDS at 20°C for 15 min and once in 0.5 × SSC with 0.1% SDS at 55°C for 30 min followed by exposure to X-ray film at −70°C for 72 h.

### Results

Treatment of both these fibroblast strains with subtoxic concentrations of the anti-oestrogen tamoxifen, or its chlorinated derivative, toremifene, resulted in a significant induction of receptor-reactive TGF-beta (Table I). Little or no induction was observed with oestradiol, three progestogens (MPA, R5020 and gestodene), dihydrotestosterone or dexamethasone. Flow 9000 cells showed a 27-fold induction of TGF-beta in response to tamoxifen, with toremifene being approximately half as effective. The induction observed in Flow 2002 cells in response to tamoxifen was approximately 5-fold over control levels, indicating that the inductive response was not restricted to a single cell strain.

Using a sensitive ligand binding technique and an immunoassay technique, the two fibroblast strains were examined for the presence of the ER protein, with the ER + ve breast cancer line MCF-7 used as a positive control.

No ER was detectable in the fibroblasts using either of these methods. Figure 1 illustrates that both fibroblast strains were devoid of any measurable ER by ligand binding using the affinity chromatography technique (detection limit 0.2 fmol of receptor per mg of soluble cellular protein), while the MCF-7 cells showed the expected high affinity intracellular receptor. Similarly, both of the fibroblast strains also failed to show any immunoreactive ER when assayed with an immunoassay kit from Abbott Laboratories, (detection limit 1.5 fmol of receptor per mg of soluble cellular protein, data not shown). Northern analysis of poly-A$^+$ selected mRNA from both of the fibroblast strains also failed to show any ER mRNA although a positive signal was obtained for poly-A$^+$ mRNA prepared from MCF-7 cells (data not shown). These observations suggest that the induction of TGF-beta might be mediated by a novel mechanism that does not involve the conventional ER. Further evidence in support of this is provided by the data in Table II. Oestradiol, used at a concentration (100 nM) that should fully abolish the binding of 500 nM tamoxifen to any ER that might be present, due to the much lower relative binding affinity of the antagonist for ER (Sutherland, 1981) causes less than a 50% reduction in the amount of TGF-beta secreted in response to tamoxifen. The reduction in TGF-beta secretion that is observed is probably due to a non-specific metabolic effect caused by the use of such a supraphysiological oestradiol concentration. By contrast, the induction of TGF-beta by anti-oestrogens in MCF-7 cells was fully reversible by oestradiol, consistent with an ER-mediated induction. Interestingly, in the present work, the putative active metabolite of tamoxifen, 4-hydroxy-tamoxifen (Jordan *et al.*, 1977), was no more potent than the parent compound in inducing TGF-beta (Table II). ICI 164,384, which is a pure oestrogen antagonist devoid of any agonist activity (Wakeling & Bowler, 1987), although less effective than tamoxifen, also caused a significant induction (Table II). Most cells in culture secrete TGF-beta in a biologically latent

**Figure 1** Scatchard analysis of oestrogen receptor in MCF-7, Flow 2002 and Flow 9000 cells by affinity immobilisation on Cibacron Blue F3GA-Sepharose 6B.

**Table I** Total TGF-beta secretion rates for the Flow 2002 and Flow 9000 cells. Secretion rates are presented as ng per $10^6$ producer cells per 48 h in monolayer culture, and are the means ± the standard deviations of triplicate estimations from duplicate experiments. The relative inductions are normalised to the ethanol vehicle control which were set at 1.0.

| | *FLOW 2002* | | *FLOW 9000* | |
|---|---|---|---|---|
| | *TGF-beta (ng $10^{-6}$ × cells 48 $h^{-1}$)* | *Rel. In.*[a] | *TGF-beta (ng $10^{-6}$ × cells 48 $h^{-1}$)* | *Rel. In.*[a] |
| Control | 0.4 ± 0.1 | 1.0 | 0.6 ± 0.0 | 1.0 |
| 10 nM Oestradiol | 0.3 ± 0.1 | 0.8 | 1.2 ± 0.2 | 1.9 |
| 500 nM Tamoxifen | 2.0 ± 0.5 | 5.0 | 16.7 ± 5.3 | 27.4 |
| 500 nM Toremifene | 1.1 ± 0.2 | 2.7 | 6.8 ± 3.6 | 11.2 |
| 500 nM MPA | <0.3 | <0.8 | <0.4 | <0.8 |
| 500 nM R5020 | <0.3 | <0.8 | <0.4 | <0.8 |
| 500 nM Gestodene | <0.3 | <0.8 | <0.4 | <0.8 |
| 10 nM DHT | 0.5 ± 0.1 | 1.3 | 0.5 ± 0.0 | 1.0 |
| 500 nM Dexamethasone | <0.3 | <0.8 | 0.5 ± 0.0 | 0.9 |

[a]*Rel. In.* = relative induction.

**Table II** Total TGF-beta secretion rates for Flow 9000 cells and the percentage of this as the active peptide. Secretion rates are presented in ng per $10^6$ producer Flow 9000 cells per 48 h in monolayer culture, and are the means ± the standard deviations of triplicate estimations of two separate experiments. The relative inductions are normalised to the ethanol vehicle control which was set at 1.0

| | *TGF-beta secreted ng $10^{-6}$ × cells 48 $h^{-1}$* | *Rel. In.*[a] | *Active TGF-beta (% of total)* |
|---|---|---|---|
| Control | 0.8 ± 0.3 | 1.0 | N.D. |
| | 0.9 ± 0.3 | 1.1 | |
| 500 nM Tamoxifen | 20.7 ± 1.8 | 25.9 | 70 |
| | 27.9 ± 2.9 | 34.9 | |
| 500 nM Tamoxifen + | 11.8 ± 0.9 | 14.8 | N.D. |
| 100 nM Oestradiol | 10.8 ± 0.9 | 13.5 | |
| 500 nM 4-Hydroxy- | 17.3 ± 1.0 | 21.6 | 72 |
| tamoxifen | 19.7 ± 3.0 | 24.6 | |
| 500 nM ICI 164,384 | 8.8 ± 0.7 | 11.0 | 100 |
| | 9.0 ± 1.1 | 11.3 | |

[a]*Rel. In.* = relative induction.

form that is unable to bind to the cellular TGF-beta receptor (Pircher *et al.*, 1984; Lawrence *et al.*, 1984; Wakefield *et al.*, 1987). By contrast, the TGF-beta secreted by the fetal fibroblasts in response to the anti-oestrogens was 70–100% in the receptor-reactive form (Table II). To date, only two other cell systems have been shown to secrete a substantial amount of active rather than latent TGF-beta. These are MCF-7 cells treated with tamoxifen (Knabbe *et al.*, 1987) and primary cultures of mouse keratinocytes treated with retinoic acid (Glick *et al.*, 1989).

To demonstrate that the TGF-beta recovered in the conditioned media was synthesised by the fibroblasts, rather than representing TGF-beta sequestered from culture components such as serum or fibronectin and later released, cells were metabolically labelled and the media immunoprecipitated with antibodies specific for two of the known TGF-beta subtypes. The data in Figure 2 confirm that the anti-oestrogens induce *de novo* synthesis of TGF-beta. Furthermore, they show the induction is specific for TGF-beta 1, or an immunologically related species, since there is no apparent induction of TGF-beta 2. In addition, extracts of the conditioned media from treated cells inhibited the growth of CCL64 cells, and this growth inhibition could be reversed by the addition of specific anti-TGF-beta 1 but not anti-TGF beta 2 antibodies (Figure 3). A complex mixture of mitogens and other inhibitors appeared to be present in all the extracts tested, but only conditioned media from those cells treated with anti-oestrogens showed inhibition that was specifically reversible with anti-TGF-beta antibodies. This demonstrates that the TGF-beta secreted has biological activity, and confirms that it is TGF-beta 1, or a very closely related species, and not TGF-beta 2. Two other TGF-beta subtypes (TGF-beta 3 and TGF-beta 4) have recently been described (Ten Dijke *et al.*, 1988; Jakowlew *et al.*, 1988). While our TGF-beta 1 antibodies show less than 1% cross-reactivity with TGF-beta 2 and TGF-beta 3 (our unpublished data), the extent of cross-reactivity with TGF-beta 4 cannot be determined until the corresponding protein becomes available. However, since the cDNA for TGF-beta 4 encodes a protein with no signal peptide, it is highly unlikely that the secreted peptide we observed is TGF-beta 4 rather than TGF-beta 1. Northern analysis showed no significant increase in TGF-beta 1 mRNA in cells treated with tamoxifen, 4-hydroxytamoxifen or ICI 164,384 compared with control cells (Figure 4). Indeed, a slight decrease in message levels was observed with ICI 164,384. Thus the mechanism of TGF-beta 1 induction by anti-oestrogens in these cells appears to be post-transcriptional. The induction of TGF-beta by anti-oestrogens in MCF-7 cells was also suggested to be post-transcriptional, although in that system induction appeared to be mediated by the classical ER (Knabbe *et al.*, 1987) so the detailed mechanisms must differ between the two systems.

**Figure 2** Immunoprecipitation of metabolically labelled TGF-beta 1 and TGF-beta 2 in the conditioned media from Flow 9000 cells. Drug treatments are indicated above the figure panels. The antibody specificity was tested by preincubation with 250 ng of either TGF-beta 1 or TGF-beta 2 as a blocking peptide. Drug concentrations are described in Materials and methods.

**Figure 3** CCL64 bioassay for TGF-beta with antibody reversal in a 1:250 dilution of the Flow 9000 conditioned media. Full details are provided in Materials and methods.

**Figure 4** Northern analysis of total cellular RNA from drug-treated Flow 9000 cells, hybridised to a specific insert derived from the TGF-beta 1 cDNA.

### Discussion

We have demonstrated for the first time an unequivocal biological action of anti-oestrogens despite the apparent lack of classical ER. The fetal fibroblasts used were devoid of this receptor by four distinct criteria: absence of functional protein in a sensitive ligand binding assay; absence of immunoreactive protein; lack of complete reversibility of the anti-oestrogen effect by high concentrations of oestradiol; and lack of any specific mRNA for ER. However, we cannot totally exclude the possibility that there exist in these cells a very small number of ER molecules that are beyond the detection limits of the methods used in our experiments.

Since the oestrogen antagonist ICI 164,384 induces TGF-beta, but does not have the structural requirements for binding to the anti-oestrogen binding site characterised by Watts *et al.* (1984), the mechanism of TGF-beta induction by anti-oestrogens does not appear to involve these sites. We anticipate that a novel binding site may be involved, although the structural dissimilarity of tamoxifen and ICI 164,384 suggest that these two drugs are unlikely to act via a common receptor, unless it were closely related to the conventional ER. This might be one of the many so-called 'orphan receptor' cDNAs that have recently been identified on the basis of their extensive sequence homology to the human ER (Giguere *et al.*, 1988; Murphy & Dotzlaw, 1989). The demonstration that pharmacological agents such as anti-oestrogens can induce the synthesis of an epithelial growth inhibitor by stromal cells opens a new horizon in the therapy and prevention of epithelial cancers.

### References

DANIELPOUR, D., DART, L.L., FLANDERS, K.C., ROBERTS, A.B. & SPORN, M.B. (1989). Immunodetection and quantitation of the two forms of transforming growth factor beta secreted by cells in culture. *J. Cell Physiol.*, **138**, 79.

EARLY BREAST CANCER TRIALLISTS COLLABORATIVE GROUP. (1988). Effects of adjuvant tamoxifen and of cytotoxic therapy on mortality in early breast cancer: An overview of 61 randomised trials among 28,896 women. *N. Engl. J. Med.*, **319**, 1681.

FEINBERG, A.P. & VOGELSTEIN, B. (1983). A technique for radiolabelling DNA restriction endonuclease fragments to a high specific activity. *Analyt. Biochem.*, **132**, 6.

GIGUERE, V., YANG, N., SEGUI, P. & EVANS, R. (1988). Identification of a new class of steroid hormone receptors. *Nature*, **331**, 91.

GLEIBER, W.E. & SCHIFFMAN, E. (1984). Identification of a chemoattractant for fibroblasts produced by human breast carcinoma cell lines. *Cancer Res.*, **44**, 3398.

GLICK, A.B., FLANDERS, K.C., DANIELPOUR, D., YUSPA, S.H. & SPORN, M.B. (1989). Retinoic acid induces transforming growth factor beta 2 in cultured keratinocytes and mouse epidermis. *Cell Regulation*, in press.

GREEN, B. & LEAKE, R.E. (1987). *Steroid Hormones – A Practical Approach*. IRL Press: Oxford.

HAGGIE, J.A., SELLWOOD, R.A., HOWELL, A., BIRCH, J.M. & SCHOR, S.L. (1987). Fibroblasts from relatives of patients with hereditary breast cancer show foetal-like behavior *in vitro*. *Lancet*, **i**, 1455.

HORGAN, K., JONES, D.L. & MANSEL, R.E. (1987). Mitogenicity of human fibroblasts *in vivo* for human breast cancer cells. *Br. J. Surgery*, **74**, 227.

IQBAL, M.J., CORBISHLEY, T.P., WILKINSON, M.L. & WILLIAMS, R. (1985). A microassay for the determination of the binding parameters of oestrogen and androgen receptors employing affinity immobilisation on Cibacron Blue F3GA-Sepharose 6B. *Analyt. Biochem.*, **144**, 79.

JAKOWLEW, S.B., DILLARD, P.B., SPORN, M.B. & ROBERTS, A.B. (1988). Complementary deoxyribonucleic acid cloning of a novel-transforming growth factor beta messenger ribonucleic acid from chick embryo chondroytes. *Mol. Endocrinol.* **2**, 1186.

JORDAN, V.C., ROWSBY, L., DIX, C.J. & PRESTWICH, G. (1977). Dose-related effects of non-steroidal antioestrogens and oestrogens on the measurement of cytoplasmic oestrgen receptors in the rat and mouse uterus. *J. Endocrinol.*, **75**, 305.

KNABBE, C., LIPPMAN, M., WAKEFIELD, L.M., DERYNK, R. & DICKSON, R.B. (1987). Evidence that transforming growth factor beta is a hormonally regulated negative growth factor in human breast cancer cells. *Cell*, **48**, 417.

KONDAIAH, P., VAN OBBERGHEN-SCHILLING, E., LUDWIG, R.L., DHAR, R., SPORN, M.B. & ROBERTS, A.B. (1981). cDNA cloning of porcine transforming growth factor beta 1 mRNAs. Evidence for alternate splicing and polyadenylation. *J. Biol. Chem.*, **263**, 18313.

KORENMAN, S.G. & DUKES, B.A. (1970). Specific estrogen binding by the cytoplasm of human breast carcinoma. *Endocrinology*, **87**, 1119.

LAEMMLI, U.K. (1970). Cleavage of structural proteins during the assembly of the head of bacteriophage T4. *Nature*, **277**, 680.

LAWRENCE, D.A., PIRCHER, R., KRYCEVE-MARTINERIE, C. & JULLIEN, P. (1984). Normal embryo fibroblasts release transforming growth factors in a latent form. *J. Cell Physiol.*, **121**, 184.

MANIATIS, T., FRISCH, E.F. & SAMBROOK, J. (1983). *Molecular Cloning: A Laboratory Manual*. Cold Spring Harbor: New York.

MEDICAL RESEARCH COUNCIL SCOTTISH TRIALS OFFICE (1988). Adjuvant tamoxifen in the management of operable breast cancer: The Scottish trial. *Lancet*, **ii**, 171.

MURPHY, L.C. & DOTZLAW, M. (1989). Variant estrogen receptor mRNA species detected in human breast cancer biopsy samples. *Mol. Endocrinol.*, **3**, 687.

NOLVADEX, ADJUVANT TRIAL ORGANISATION. (1987). Controlled trial of tamoxifen as a single adjuvant agent in the management of early breast cancer. *Br. J. Cancer*, **57**, 608.

PIRCHER, R., LAWRENCE, D.A. & JULLIEN, P. (1984). Latent beta-transforming growth factor in non-transformed and Kirsten virus-transformed normal rat kidney cells, clone 49F. *Cancer Res.*, **44**, 5538.

SKIDMORE, J.R., WALPOLE, A.L. & WOODBURN, J. (1972). Effect of some triphenylethylenes on oestradiol binding *in vitro* to macromolecules from the uterus and anterior pituitary. *J. Endocrinol.*, **52**, 289.

SUTHERLAND, R.L. (1981). Estrogen antagonists in the chick oviduct: antagonist activity of eight synthetic triphenylethylene derivatives and their interactions with cytoplasmic and nuclear estrogen receptors. *Endocrinology*, **109**, 2061.

TEN DIJKE, P., HANSEN, P., IWATA, K.K., PIELER, C. & FOULKES, J.G. (1988). Identification of another member of the transforming growth factor type beta family. *Proc. Natl Acad. Sci. USA*, **85**, 4715.

WAKEFIELD, L.M., SMITH, D.M., MATSUI, T., HARRIS, C. & SPORN, M.B. (1987). Distribution and modulation of the cellular receptor for transforming growth factor beta. *J. Cell Biol.*, **105**, 965.

WAKELING, A.E. & BOWLER, J. (1987). Steroidal pure antioestrogens. *J. Endocrinol.*, **112**, R7.

WATTS, C.K.W., MURPHY, L.C. & SUTHERLAND, R.L. (1984). Microsomal binding sites for non-steroidal antioestrogens in MCF-7 human mammary carcinoma cells. Demonstration of high affinity and narrow specificity for basic ether derivatives of triphenylethylene. *J. Biol. Chem.*, **259**, 4223.